# Plasma Retinoid Concentrations Are Altered in Pregnant Women

**DOI:** 10.3390/nu14071365

**Published:** 2022-03-25

**Authors:** Lindsay C. Czuba, Emily E. Fay, Jeffrey LaFrance, Chase K. Smith, Sara Shum, Sue L. Moreni, Jennie Mao, Nina Isoherranen, Mary F. Hebert

**Affiliations:** 1 Department of Pharmaceutics, School of Pharmacy, University of Washington, Seattle, WA 98195, USA; lczuba@uw.edu (L.C.C.); lafraj@uw.edu (J.L.); hms520@uw.edu (S.S.); ni2@uw.edu (N.I.); 2 Department of Obstetrics and Gynecology, School of Medicine, University of Washington, Seattle, WA 98195, USA; efay@uw.edu (E.E.F.); leesue@uw.edu (S.L.M.); jmao2@uw.edu (J.M.); 3 Department of Pharmacy, School of Pharmacy, University of Washington, Seattle, WA 98195, USA; smith406@uw.edu

**Keywords:** vitamin A, retinoid, pregnancy, postpartum, retinol-binding protein 4 (RBP4), transthyretin (TTR)

## Abstract

Vitamin A is vital to maternal–fetal health and pregnancy outcomes. However, little is known about pregnancy associated changes in maternal vitamin A homeostasis and concentrations of circulating retinol metabolites. The goal of this study was to characterize retinoid concentrations in healthy women (*n* = 23) during two stages of pregnancy (25–28 weeks gestation and 28–32 weeks gestation) as compared to ≥3 months postpartum. It was hypothesized that plasma retinol, retinol binding protein 4 (RBP4), transthyretin and albumin concentrations would decline during pregnancy and return to baseline by 3 months postpartum. At 25–28 weeks gestation, plasma retinol (−27%), 4-oxo-13-*cis*-retinoic acid (−34%), and albumin (−22%) concentrations were significantly lower, and *all-trans*-retinoic acid (+48%) concentrations were significantly higher compared to ≥3 months postpartum in healthy women. In addition, at 28–32 weeks gestation, plasma retinol (−41%), retinol binding protein 4 (RBP4; −17%), transthyretin (TTR; −21%), albumin (−26%), 13-*cis*-retinoic acid (−23%) and 4-oxo-13-*cis*-retinoic acid (−48%) concentrations were significantly lower, whereas plasma *all-trans*-retinoic acid concentrations (+30%) were significantly higher than ≥3 months postpartum. Collectively, the data demonstrates that in healthy pregnancies, retinol plasma concentrations are lower, but *all-trans*-retinoic acid concentrations are higher than postpartum.

## 1. Introduction

Vitamin A is a nutrient critical for reproduction, fetal development, growth, vision, and transcriptional regulation of gene expression [1]. Dietary retinyl esters and beta-carotene are the predominant sources of vitamin A (retinol). Retinol circulates bound to the retinol binding protein 4 (RBP4) and can complex with transthyretin (TTR) [2]. In healthy males and females from high income countries, circulating retinol concentrations have been reported to be between 1–2 μM and to vary less than 20% [3,4,5], while various conditions including vitamin A deficiency [6], chronic kidney disease [3], obesity [7], and pregnancy [8] may be associated with altered circulating concentrations or variability in retinol. Vitamin A bioactivity is attributed to the metabolite *all-trans*-retinoic acid (*at*RA), while other metabolites such as 13-*cis*-retinoic acid (13*cis*RA), and the downstream metabolite of 13*cis*RA, 4-oxo-13-*cis*-retinoic acid (4oxo13*cis*RA) are also present in circulation and tissues in humans [2]. These metabolites circulate at lower concentrations than retinol [9] and bind to albumin [1,10] such that <0.001% circulates as the free form [11].

During pregnancy, the transplacental transfer of maternal retinoids is essential for fetal development [8,12,13]. Unbound *at*RA and 13*cis*RA cross the placental barrier from maternal circulation, but CYP26 enzymes regulate exposure to *at*RA in individual fetal organs, thereby protecting the fetus from teratogenic concentrations [14,15]. In addition, retinol crosses the placental barrier without maternal RBP4, however expression of fetal RBP4 is needed for retinol transfer from the placenta to the fetus [13,16]. To maintain adequate circulating concentrations of maternal retinol and support maternal retinoic acid signaling in extrahepatic tissues, hepatic stores of vitamin A can be used as a source for circulating retinol in lieu of adequate intake. However, a lack of adequate whole-body stores leads to deficiency and is detrimental to fetal development and pregnancy outcomes [8,17]. Maternal vitamin A deficiency, which is defined as plasma concentrations < 0.7 μM, is associated with night blindness, maternal anemia, risk for preeclampsia, and preterm delivery [18,19]. Vitamin A ‘sufficiency’ is clinically defined by plasma retinol concentrations >1.05 μM. Vitamin A deficiency is not believed to be a major health problem in the general population within the USA, and the prevalence of vitamin A deficiency or insufficiency in non-pregnant women of childbearing age is ~3.9% [19]. However, maternal retinol concentrations may fall below 1.05 μM during pregnancy [6,20], and a proportion of pregnant women may experience periods of vitamin A insufficiency. For example, the prevalence of low retinol concentrations (0.7–1.05 μM) in healthy, well-nourished pregnant women from one New Jersey urban hospital was 17%, 18%, and 23% in the first, second and third trimesters, respectively [21]. Similarly, 41.4% of pregnant women enrolled as healthy, pregnant controls from Nebraska had insufficient retinol concentrations at delivery, of which 10% were clinically deficient [20].

Retinol is not known to circulate unbound from RBP4 in plasma [22]. However, it is unclear if a decrease in retinol during pregnancy correlates with a decrease in circulating RBP4 concentrations as data is inconsistent between studies [22,23,24,25]. In one study in healthy, pregnant women, serum RBP4 was lower at 26–28 weeks and 36–37 weeks gestation in comparison to 11–12 weeks gestation [24]. In contrast, a second study found lower fasting RBP4 concentrations at 14–16 weeks gestation in comparison to 30–32 weeks gestation [25]. These discrepancies may be due to methodological differences, gestational time differences, or population differences. Elevated RBP4 concentrations have been implicated in various pregnancy complications including preeclampsia, although the data remains controversial as there are inconsistencies noted between studies [26]. It is not known whether the maternal changes in retinol and RBP4 correspond to increased formation of vitamin A [22]. To date, only a single study investigated if the bioactive metabolite, *at*RA, and its isomer 13*cis*RA are altered during pregnancy and demonstrated higher plasma *at*RA concentrations at the time of delivery in comparison to non-pregnant controls. In addition, 13*cis*RA concentrations were lower at the time of delivery in comparison to 8–12 weeks gestation and to non-pregnant controls [27]. This would indicate that circulating concentrations of retinol may not be a sensitive biomarker for predicting fetal exposure to maternal *at*RA or correlating retinoid signaling to pregnancy outcomes. During pregnancy, albumin concentrations decrease [28], such that an increase in the free fraction of *at*RA may also regularly occur. This is critical at the early stages of pregnancy where too high or too low concentrations of retinoic acids may be detrimental, potentially resulting in miscarriage or fetal abnormalities. There is limited dose-response data in pregnant women to understand how endogenous retinoic acid concentrations, and specifically the free fraction, change throughout pregnancy, impact maternal health, or predict pregnancy outcomes. Thus, there is a critical need to establish if retinoid concentrations are altered in healthy pregnancies and to establish trimesters specific changes in the free concentrations of *at*RA. This data will provide much needed insight into the effects of pregnancy on maternal retinoid concentrations and potentially identify an area for future research (i.e., vitamin A intake during pregnancy in the USA).

In the current study, we hypothesized that plasma retinol, retinol binding protein 4 (RBP4), transthyretin and albumin concentrations would be lower during pregnancy in comparison to 3 months postpartum. Retinoid concentrations were quantified from plasma samples obtained from healthy pregnant women 25–28 weeks gestation, 28–32 weeks gestation, and ≥3 months postpartum using LC-MS/MS to determine if vitamin A homeostasis is altered in human pregnancy.

## 2. Materials and Methods

### 2.1. Materials

Optima LC/MS grade acetonitrile, water, methanol, and formic acid were from Thermo Fischer Scientific (Waltham, MA, USA). Human serum (DC Mass Spect Gold MSG 4000) was purchased from Golden West Biologics (Temecula, CA, USA). *at*RA-d_5_, 13*cis*RA-d_5_ were purchased from Cayman Chemical (Ann Arbor, MI, USA) and retinol-d_6_ was purchased from Cambridge Isotopes Laboratories (Tewksbury, MA, USA). *at*RA, 13*cis*RA and *all-trans*-retinol were purchased from MilliporeSigma (Burlington, MA, USA) and 4oxo*at*RA, 4oxo13*cis*RA, and 4oxo13*cis*RA-d_3_ were purchased from Santa Cruz Biotechnology (Dallas, TX, USA).

### 2.2. Study Participants

Healthy, pregnant (singleton pregnancies), 18–50 years old women were enrolled in this study. All subjects provided written informed consent. Exclusion criteria included a history of obesity (BMI > 30 kg/m^2^ based on pre-pregnancy weight), diabetes, kidney disease, or liver disease. Additionally, treatment for mental illness, vitamin A supplementation other than prenatal vitamins, or any current illness involving a fever or cough were exclusionary. Vitamin A dietary intake was calculated based on participant food logs that were recorded for the three days prior to each study visit. Each food item recorded was converted to its corresponding amount of vitamin A (IU) using a database of nutritional content provided by Fooducate LTD (San Francisco, CA, USA). The average three-day intake of vitamin A was calculated in excel and data are presented as the amount of vitamin A (IU). Unit conversion factors were obtained from the USDA Dietary Supplement Ingredient Database (DSID), where 1 IU is equal to 0.3 μg retinol and 1 μg retinol is equivalent to 1 μg retinol activity equivalents (RAE). The study was approved by the Institutional Review Board at the University of Washington (STUDY00001620, approved 28 March 2017) and conducted in accordance with the Declaration of Helsinki principles.

### 2.3. Quantification of Retinoids in Plasma

Blood samples were obtained from participants 30 min after breakfast into K2 EDTA vacutainer tubes, light-protected (tubes were wrapped with aluminum foil and laboratory lights cover with UV filters), and plasma isolated following centrifugation at 3000× *g* for 10 min at 4 °C. Plasma was stored at −80 °C in amber vials. Endogenous retinoids were quantified from human plasma using previously established ultra-high-performance liquid chromatography mass spectrometry (UHPLC-MS/MS) methods [9,29]. The standard curve contained *at*RA (1–20 nM), 13*cis*RA (1–20 nM), 4oxo13*cis*RA (2–40 nM), 4oxo*at*RA (1–20 nM), and retinol (250–2500 nM). Independent quality control (QC) samples were prepared in triplicate at three concentrations within the standard curve range. For analysis, 60 μL of light protected human plasma samples, standard curve samples, and QC samples were protein precipitated with 120 μL of ice-cold acetonitrile containing 50 nM 4oxo13*cis*RA-d_3_, *at*RA-d_5_, 13*cis*RA-d_5_, and 750 nM retinol-d_6_. Samples were gently mixed by pipetting and plates centrifuged at 3000× *g* for 40 min at 4 °C. The supernatant was transferred to a new 96-well plate followed by a second centrifugation step at 3000× *g* for an additional 30 min at 4 °C. The final cleared supernatant was transferred to a 96-well plate for UHPLC-MS/MS analysis of *at*RA, 13*cis*RA, and 4oxo13*cis*RA. For the analysis of retinol, a portion of the cleared supernatant was transferred to a 96-well plate and diluted 1:20 with acetonitrile before analysis. Sample preparation was performed on ice and under yellow-red lights.

All UHPLC-MS/MS parameters and chromatography were as previously described [9,29]. Plasma retinoids were separated using an Agilent 1290 Infinity II UHPLC (Santa Clara, CA, USA) coupled to an Ascentis Express RP Amide column (2.7 μm; 150 mm × 2.1 mm) and analytes detected on an AB Sciex 6500 (RA and metabolites) or 5500 (retinol) qTrap Q-LIT mass spectrometer (Foster City, CA, USA). The mass spectrometer was operated in positive ion APCI mode. Analyte peaks were integrated using MultiQuant 2.1.1 (Sciex) and peak area ratios were quantified against the global fit of the two standard curves analyzed (weighted 1/x). For run acceptance, at each QC concentration, at least 2/3 of QC samples were within 15% of the nominal concentration in accordance with bioanalytical guidelines [30].

### 2.4. Quantification of Retinol-Binding Protein 4 (RBP4) and Transthyretin (TTR) in Plasma

Using a Human RBP4 Quantikine ELISA Kit (R & D Systems, Minneapolis, MN, USA), plasma retinol-binding protein 4 (RBP4) was quantified as previously described from light protected samples according to manufacturer’s recommendations [31]. The standard curve was plotted as the log(concentration) vs. log(optical density). Human plasma transthyretin was quantified using a human TTR ELISA kit (catalog no. KA0495) from Abnova (Taipei City, Taiwan). Light-protected plasma samples were prepared according to the manufacturer’s recommendations. Samples were quantified against a standard curve fit to a 4-parameter logistic curve in GraphPad Prism 9.1 (San Diego, CA, USA), as recommended by the manufacturer.

### 2.5. Statistical Methods

Data are expressed as geometric mean and interquartile range (25th percentile, 75th percentile), unless otherwise indicated. Statistical comparisons were performed using GraphPad Prism 9.1 (San Diego, CA, USA). To determine if the average vitamin A intake for each subject was statistically different between study days, a repeated measures one-way ANOVA and Šídák’s multiple comparison test were performed. Study Day 1 (SD1) and 2 (SD2) samples were compared to each participant’s Study Day 3 (SD3) sample using a Friedman Test (paired, non-parametric analysis) and Dunn’s post hoc analysis. *p*-values were corrected for multiple comparisons with significance at *p* < 0.05. Absolute *p*-values are reported.

## 3. Results

### 3.1. Human Subjects

Twenty-three pregnant women were enrolled prior to 28 weeks gestation and study characteristics are described in Table 1. Participants were overall healthy with no history of obesity (pre-pregnancy BMI 25.5 ± 3.1 kg/m^2^ (mean ± SD)), diabetes, liver, or kidney disease. Three participants had mild anemia and two reported hypothyroidism (ongoing treatment). One participant was diagnosed with each of the following: hyperemesis gravidarum (resolved prior to study participation), placenta previa, preeclampsia (resolved postpartum) and gestational hypertension. For SD3, 91% of participants were breastfeeding.

**Figure 1:**
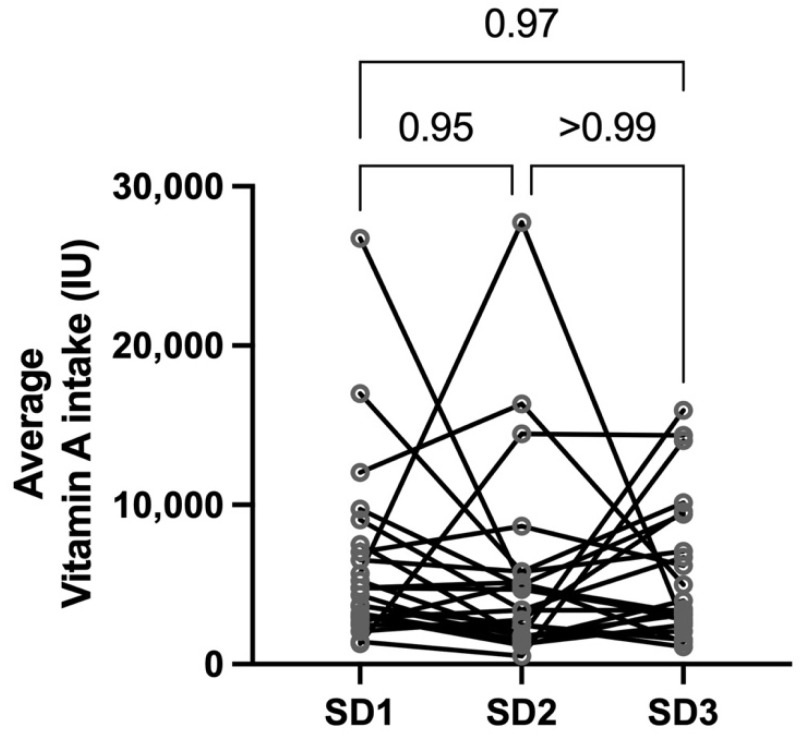
The average vitamin A intake (IU) for each participant was calculated based on self-reported food logs for the three days prior to SD1, SD2, and SD3 visits and were not significantly different between visits.

Prenatal vitamin supplements were taken by 87% of participants at SD1 (25–28 weeks gestation) and 57% reported supplementation in the postpartum period. No participants supplemented with vitamins containing vitamin A between SD2 and SD3 in accordance with the study. The geometric mean daily dietary intake of Vitamin A was 4790 IU (IQR 2956; 7280 IU), 3620 IU (IQR 1776 IU; 5463 IU), and 4372 IU (IQR 2689 IU; 8240 IU) on SD1, SD2 and SD3, respectively. There was no statistical difference in the vitamin A intake on SD1 compared to SD2 (*p* = 0.95), or on either SD1 (*p* = 0.97) or SD2 (*p* > 0.99) when compared to the matched SD3 (postpartum) (Figure 1). However, four (17%) and nine (39%) participants on SD1 and SD2, respectively, did not meet the recommended intake of vitamin A of 770 μg of retinol equivalents [12] (or ~2564 IU vitamin A). On SD1, only 1 of the 4 participants with low vitamin A intake reported no prenatal vitamin use. In addition, 57% of lactating participants did not meet the recommended daily intake of 1300 μg of retinol equivalents [12] or 4329 IU.

### 3.2. Plasma Retinoids during Normal Healthy Pregnancies

**Figure 2:**
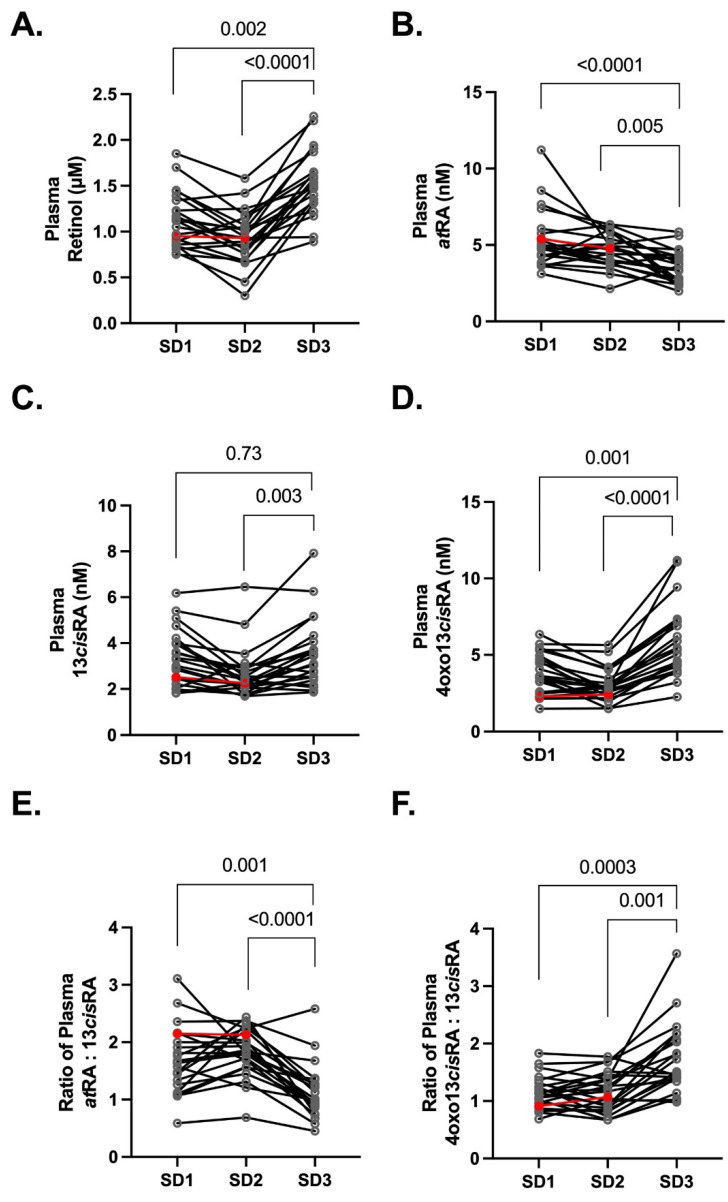
The plasma retinoid ((**A**) retinol (**B**) *at*RA (**C**) 13*cis*RA (**D**) 4oxo13*cis*RA) concentrations are altered in normal healthy pregnancies (*n* = 23) at 25–28 weeks gestation (SD1) and 28–32 weeks gestation (SD2) compared to 3–4 months PP (SD3). The ratio of (**E**) *at*RA to 13*cis*RA was higher during pregnancy while the ratio of (**F**) 4oxo13*cis*RA to 13*cis*RA was lower during pregnancy. In each analysis, one subject was excluded from the statistical comparison due to insufficient sample quantity in SD3 (red symbols).

During SD1 (25–28 weeks gestation) and SD2 (28–32 weeks gestation), retinoid concentrations were altered relative to the non-pregnant control study (SD3, ≥3 months postpartum). Plasma retinol concentrations (Figure 2A) were lowest on SD2 (0.9 μM (IQR 0.8, 1.1 μM) *p* < 0.0001), followed by SD1 (1.1 μM (IQR 0.9, 1.3 μM) *p* = 0.002) and SD3 (1.5 μM (IQR 1.3–1.7 μM)). Five pregnant participants met the criteria for Vitamin A deficiency based on plasma retinol concentrations < 0.70 μM in SD2 and 43% and 48% of participants had insufficient retinol concentrations (clinically defined as >0.70 μM but <1.05 μM) on SD1 and SD2, respectively. Despite the high frequency of low retinol concentrations, there was no correlation between retinol concentrations and daily intake on either SD1 (non-parametric Spearman r = 0.16; *p* = 0.5) or SD2 (non-parametric Spearman r = 0.09; *p* = 0.7). The low vitamin A status was transient, as >90% participants returned to vitamin A sufficient status by ≥3 months postpartum. Notably, the retinoid concentrations on SD3 were higher than during pregnancy despite the high prevalence of lactating women not meeting the recommended daily intake of vitamin A. Similar to SD1 and SD2, there was no correlation with retinol concentrations or dietary intake during SD3 postpartum visit (non-parametric Spearman r = 0.06, *p* = 0.8).

Unlike retinol, the plasma *at*RA concentrations were higher during pregnancy in comparison to postpartum (Figure 2B), with the geometric mean concentrations of 5.1 nM (IQR 4.3, 5.6 nM) *p* < 0.0001, 4.5 nM (IQR 3.9, 5.2 nM) *p* = 0.005, and 3.4 nM (IQR 2.7, 4.3 nM) measured on SD1, SD2, and SD3 respectively. The concentrations of 13*cis*RA (Figure 2C) were significantly lower on SD2 (geometric mean 2.5 nM (IQR 2.1, 2.9 nM) *p* = 0.003) in comparison to the paired SD3 concentrations (geometric mean 3.3 nM (IQR 2.5, 4.0 nM)), but the concentrations on SD1 (geometric mean 3.2 nM (IQR 2.4, 4.0 nM)) were similar to SD3. The 13*cis*RA metabolite 4oxo13*cis*RA concentrations were significantly lower on SD1 (geometric mean 3.6 nM (IQR 2.6, 4.8 nM) *p* = 0.001) and SD2 (geometric mean 2.8 nM (IQR 2.3, 3.3 nM) *p* < 0.0001) in comparison to SD3 (geometric mean 5.4 nM (IQR 4.3, 7.1 nM)) (Figure 2D). The ratio of *at*RA to 13*cis*RA was higher during SD1 (geometric mean ratio 1.6 (IQR 1.3, 2.1); *p* = 0.001) and SD2 (geometric mean ratio 1.8 (IQR 0.9, 1.4); *p* < 0.0001) in comparison to SD3 (geometric mean ratio 1.0 (IQR 0.9, 1.3)) (Figure 2E), while the ratio of 4oxo13*cis*RA to 13*cis*RA was lower SD1 (geometric mean ratio 1.1 (IQR 1.0, 1.3); *p* = 0.0003) and SD2 (geometric mean ratio 1.1 (IQR 0.9, 1.4); *p* = 0.001) in comparison to SD3 (geometric mean ratio 1.6 (IQR 1.4, 2.1) (Figure 2F).

### 3.3. Plasma Concentrations of Retinol Binding Protein 4 (RBP4) and the Binding Partner Transthyretin (TTR)

**Figure 3:**
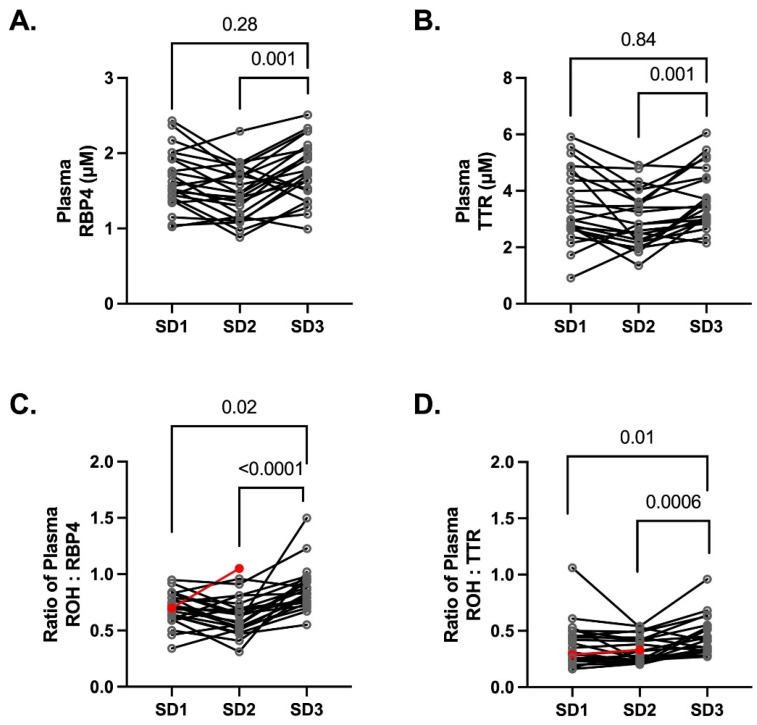
The plasma (**A**) RBP4 and (**B**) TTR concentrations at 25–28 weeks gestation (SD1), 28–32 weeks gestation (SD2), and 3–4 months PP (SD3) were measured from 23 healthy pregnant women. The ratio of (**C**) retinol to RBP4 and (**D**) retinol to TTR were lower on both SD1 and SD2 in comparison to SD3. In panels **C**,**D**, one subject had insufficient sample to measure SD3 retinol concentrations (red symbols) and was excluded from the statistical comparison (*n* = 22 pregnant women).

In circulation, retinol is bound to RBP4 and complexed with TTR [32]. RBP4 and TTR were measured in plasma to determine if changes in the total retinol concentrations were associated with changes in retinol binding in circulation (Figure 3). The geometric mean (IQR) of RBP4 was 1.1 μM (IQR 0.9, 1.3 μM), 1.4 μM (IQR 1.2, 1.7 μM), and 1.7 μM (IQR 1.5, 2.1 μM) measured on SD1, SD2, and SD3 respectively. During pregnancy, RBP4 (Figure 3A) plasma concentrations were lower on SD2 (*p* = 0.001) compared to paired SD3 samples, while SD1 concentrations were similar (*p* = 0.28) to SD3. Additionally, plasma TTR concentrations (Figure 3B) were lower on SD2 (geometric mean 2.8 μM (IQR 2.2, 3.6 μM); *p* = 0.001) in comparison to paired SD3 samples (geometric mean 3.6 μM (IQR 2.0, 4.4 μM), while SD1 concentrations were similar (geometric mean 3.2 μM (IQR 2.7, 4.5 μM); *p* = 0.84) to SD3. As expected, retinol concentrations in plasma were about the same as RBP4 and the geometric mean of the retinol to RBP4 concentration ratio was 0.7 (IQR 0.6, 0.8) on SD1, 0.6 (IQR 0.5, 0.7) on SD2, and 0.9 (IQR 0.8, 0.9) in the non-pregnant state (SD3; postpartum) (Figure 3C). On the non-pregnant postpartum control day (SD3; postpartum), the ratio of retinol to TTR was 0.4 (IQR 0.3, 0.5), while during pregnancy the geometric mean ratio on SD1 and SD2 was 0.3 (IQR 0.3, 0.5) and 0.3 (IQR 0.2, 0.4), respectively (Figure 3D). The ratio of retinol to RBP4 and retinol to TTR was lower on both pregnancy study days (Figure 3C,D) in comparison to the paired SD3 concentrations.

### 3.4. Free atRA Concentrations during Pregnancy and Postpartum

**Figure 4:**
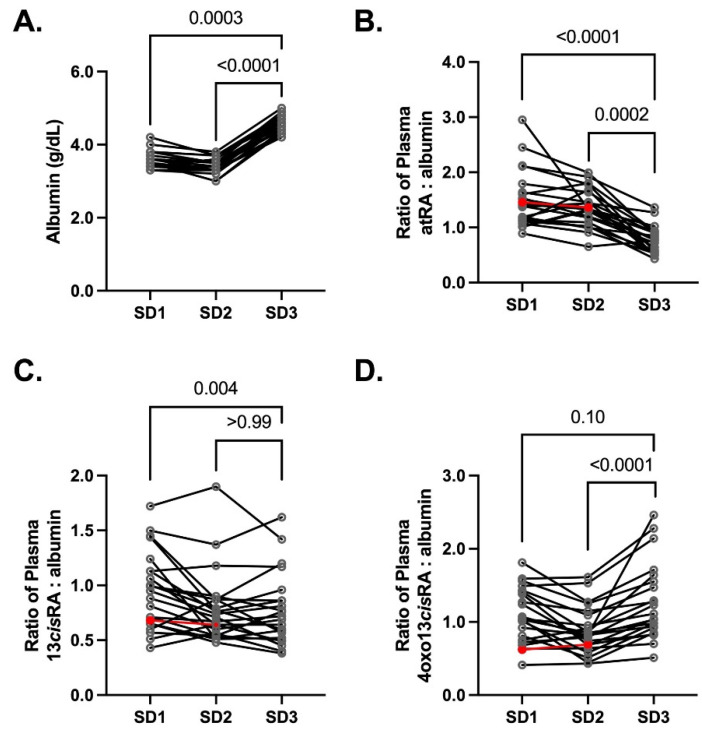
Plasma (**A**) albumin was measured on each study visit during 25–28 weeks gestation (SD1), 28–32 weeks gestation (SD2), and 3–4 months PP (SD3). Plasma retinoid concentrations were normalized to the albumin concentration and shown as a ratio in (**B**–**D**). In panels **B**–**D**, one subject was excluded for statistical comparisons due insufficient sample for SD3 retinoid concentrations (red symbols) (*n* = 22 pregnant women).

In plasma, the fraction of *at*RA bound is >0.99, [11] likely bound to albumin [1,33]. Pregnancy is associated with a decrease in albumin concentrations [28]. Albumin was about 20% lower on SD1 (geometric mean = 3.6 g/dL (IQR 3.4, 3.7 g/dL) *p* = 0.0003) and 30% lower on SD2 (geometric mean = 3.4 g/dL (IQR 3.3, 3.5 g/dL) *p* < 0.0001) in comparison to SD3 (geometric mean = 4.6 g/dL (IQR 4.5, 4.8 g/dL)) in the current cohort (Figure 4A). The ratio of *at*RA/albumin in plasma was notably higher at both pregnancy time points compared with postpartum. This suggests a higher free fraction of *at*RA, which is consistent with a lower albumin concentration during pregnancy. A modest difference (*p* = 0.004) was detected with the 13*cis*RA: albumin ratio on SD1 unlike SD2 (*p* > 0.99). Since 13*cis*RA has a low extraction ratio, any change in the free fraction with lower albumin would be expected to result in higher clearance and lower total plasma concentration. Plasma 4oxo13*cis*RA concentrations were lower on SD1 and SD2 when compared to SD3 (Figure 2D), but the 4oxo13*cis*RA to albumin ratios were only significantly lower on SD2 (*p* < 0.0001). Collectively, this suggests that the free fraction of 4oxo13*cis*RA and the total concentration is altered on SD2, which can be attributed to either a lower formation or higher clearance of the metabolite.

## 4. Discussion

Over the past 10 years, few studies have investigated pregnancy associated changes to plasma Vitamin A (‘retinol’) concentrations in healthy women. To our knowledge, the current analysis is the first to quantitatively measure plasma retinol, *at*RA, 13*cis*RA, 4oxo13*cis*RA, and plasma retinoid binding proteins within the same subjects from a cohort of pregnant women. Pregnancy was associated with altered plasma retinoids and binding partners in comparison to postpartum samples, although the magnitude and direction of change was gestational age and retinoid specific.

The current data clearly demonstrate that plasma retinol concentrations are lower during pregnancy than in the postpartum period. Vitamin A homeostasis is tightly regulated to maintain steady-state retinol concentrations in circulation [2]. Yet, it has been widely reported from animal and human studies that retinol concentrations decline in late pregnancy [16,20,21,23]. In the current analysis, the pre-pregnancy vitamin A status of the participants was unknown. Although all participants were healthy, and >90% had sufficient concentrations of retinol in the postpartum period, a significant number of women did not meet the recommended daily intake of vitamin A at each study day. Surprisingly, 43% and 48% of participants between 25–28 weeks gestation and 28–32 weeks gestation presented with vitamin A insufficiency, and there was a lack of correlation between the vitamin A intake and the retinol concentrations. Our findings on the prevalence of vitamin A insufficiency from healthy, well-nourished pregnant women agree with other recent reports.

In a recent report on pregnant women from Nebraska, the median reported retinol concentration at delivery was 1.06 μM, and less than 50% of the women met the criteria for vitamin A sufficiency, while 41.4% of women had insufficient plasma retinol concentrations and 10% were either deficient or extremely deficient (<0.35 μM) [20]. Likewise, in an urban, ethnically diverse US population, the prevalence of vitamin A insufficiency and/or deficiency (<1.05 μM) at the time of delivery was 62% [34]. The higher prevalence of vitamin A insufficiency in US urban population may be due to a combination of differences in diets, third trimester sample time point, differences in demographics and/or genetic contributions [6]. In contrast to our findings, multiple large-scale investigations from pregnant women in various cities and provinces in China have found different patterns of changes during pregnancy or different prevalence of deficiency/insufficiency [35,36,37]. For instance, a recent investigation from Beijing (*n* = 31,301 women) did not see a significant difference at 14–27 weeks gestation in the concentrations of plasma retinol in comparison to 1–13 weeks of pregnancy, while the mean plasma retinol concentrations at 28–39 weeks were significantly lower than in the first trimester [38]. A second analysis involving women across 17 cities and 4 provinces in China reported higher retinol concentrations in the second trimester in comparison to the first and third, although the average gestational age for each trimester was not reported [35]. Notably, both analyses lacked a non-pregnant control or postpartum group that may be required to detect pregnancy specific effects. Irrespective of temporal differences in the patterns of the detected changes, the current and prior studies describe reproducible reduction in retinol concentrations during pregnancy which may partly be independent of vitamin A or β-carotene intake.

It is likely that pregnancy alters vitamin A metabolism and homeostasis in maternal tissues, however the mechanism(s) driving changes in plasma retinol, *at*RA, 13*cis*RA, 4oxo13*cis*RA, and binding protein concentrations are unclear and cannot be elucidated in the current analysis. Recently, a kinetic model of retinol changes was reported based on rodent tracer studies and implicated the mobilization of liver retinoids in late pregnancy [17]. The liver is a major site for vitamin A metabolism, storage, RBP4 and albumin synthesis [2]. Observed changes in the retinol to RBP4 ratio during pregnancy may reflect a decrease in RBP4 production and release from the liver or an increase in the clearance of RBP4-bound retinol during pregnancy. Plasma RBP4 concentrations may predict the risk for preeclampsia [26,39], pre-term delivery [26], and/or gestational diabetes [32]. However, there is a clear lack of knowledge regarding changes to plasma RBP4, retinol to RBP4 plasma ratio, or retinol to TTR plasma ratio between mid and late pregnancy compared to postpartum in normal pregnancy. The data here show that RBP4 is lower during 28–32 weeks gestation compared to ≥3 months postpartum. Similarly, a previous study demonstrated lower RBP4 and TTR concentrations at delivery in comparison to non-pregnant controls [23]. In contrast, one study reported that RBP4 was higher at 30 weeks of gestation compared to 6 weeks and 6 months postpartum [40]. These differences may reflect methodological differences in the sample preparation, detection method, or population differences. Notably, the ratio of retinol to RBP4 was unaffected in one recent study at delivery in comparison to non-pregnant controls [23], in contrast to data presented in the current study and in another previous work [22]. In the current analysis, the observed geometric mean of the ratio of retinol to TTR and retinol to RBP4 during pregnancy agreed with a previous report of a median RBP4 to TTR ratio of 0.4 and RBP4 to retinol ratio of 1.1 at 29 weeks (median) of pregnancy [22].

It is notable that *at*RA concentrations are higher during 25–28 weeks gestation and 28–32 weeks gestation in comparison to postpartum, while 13*cis*RA and its metabolite are lower. Higher concentrations of *at*RA during 25–28 weeks gestation and 28–32 weeks gestation may alter pregnancy and fetal complication risks. Retinoic acid concentrations are tightly regulated during embryogenesis and fetal development. Although maternal *at*RA does cross to the placenta, fetal CYP26 enzymes readily metabolize retinoic acid [13,15,16]. As *at*RA clearance is likely unchanged during pregnancy due to its high extraction in the liver and other tissues, the increase in total concentration of *at*RA is likely due to increased synthesis. It is not known if the increased maternal free fraction of *at*RA observed during 25–28 weeks gestation, presumably a result of decreased albumin concentrations and increased synthesis, correlate to increased fetal exposure or if it drives maternal signaling needs and placental-specific signaling. To our knowledge, only one other study has reported changes to retinoic acid in human pregnancy and supports our findings [27]. Furthermore, like the current analysis, correction of *at*RA concentration for albumin concentration indicated a pronounced effect of pregnancy on free *at*RA concentrations when measured at delivery in comparison to 8–12 weeks gestation, while longitudinal data from a single pregnant woman indicates elevated free fraction may occur as early as 4 months of pregnancy [27]. The significantly lower concentrations of 13*cis*RA during 28–32 weeks gestation could no longer be detected after albumin correction unlike each of the other retinoids. This agrees with its low metabolic extraction, such that a change in albumin bound 13*cis*RA would result in an increase in clearance of 13*cis*RA and decreased total concentrations without any change in unbound 13*cis*RA clearance. The lower circulating concentration of 4oxo13*cis*RA observed both during 25–28 weeks gestation and 28–32 weeks gestation relative to postpartum may be attributed primarily to a higher clearance of 4oxo13*cis*RA, rather than lower formation from 13*cis*RA.

Strengths of the study include the paired analysis performed allowing discrete changes within individuals to be detected, measurements of multiple vitamin A metabolites and binding partners in each sample, and investigation of gestational age specific effects. However, while the study is representative of the population within the University of Washington clinics, it is not representative of the entire USA population, which may have additional genetic confounders and other factors that could alter retinoid concentrations. 

## 5. Conclusions

Collectively, the current analysis provides requisite evidence of systemic changes to maternal retinoid concentrations and major binding partners. Pronounced changes in the circulating concentration of the bioactive metabolite *at*RA during mid- to late-pregnancy warrant follow-up to determine the biological significance and elucidate tissue targets sensitive to the change. In addition, future mechanistic studies are needed to determine the driving factors promoting decreased plasma retinol concentrations in pregnant women.

## Figures and Tables

**Table 1:** Study participant characteristics.

Characteristic	Value (Mean ± SD)
Women (*n* = 23)	
Age (y)	32.4 ± 3.1 ^A^
Height (cm)	161.9 ± 21.6
Pre-pregnancy weight (kg)	64.8 ± 10.7
Pre-pregnancy BMI (kg/m^2^)	25.5 ± 3.1
Race ^B^	**%**
White	70
Asian	13
Black	9
Pacific Islander	4
Mixed Asian and Caucasian	4
Study Days	Weeks (mean ± SD)
Study Day 1	26.8 ± 1.2 weeks gestation
Study Day 2	30.1 ± 1.2 weeks gestation
Study Day 3	15.3 ± 2.6 weeks postpartum
Prenatal vitamin supplementation	Frequency (%)
Study Day 1	87%
Study Day 2	0%
Study Day 3	57%
Lactating	91%

^A^ Participant information on study day 1; ^B^ Black participants were of African descent and one white subject’s ethnicity was Hispanic/Latina.

## Data Availability

The data presented in this study are available from all participants involved in the study.
